# Direction of Apparent Motion During Smooth Pursuit Is Determined Using a Mixture of Retinal and Objective Proximities

**DOI:** 10.1177/2041669520937320

**Published:** 2020-06-26

**Authors:** Masahiko Terao, Shin’ya Nishida

**Affiliations:** The research Institute for Time Studies, Yamaguchi University; NTT Communication Science Laboratories, Kyoto, Japan; Graduate School of Informatics, Kyoto University

**Keywords:** eye movements, motion, smooth pursuit, apparent motion, extraretinal motion

## Abstract

Many studies have investigated various effects of smooth pursuit on visual motion processing, especially the effects related to the additional retinal shifts produced by eye movement. In this article, we show that the perception of apparent motion during smooth pursuit is determined by the interelement proximity in retinal coordinates and also by the proximity in objective world coordinates. In Experiment 1, we investigated the perceived direction of the two-frame apparent motion of a square-wave grating with various displacement sizes under fixation and pursuit viewing conditions. The retinal and objective displacements between the two frames agreed with each other under the fixation condition. However, the displacements differed by 180 degrees in terms of phase shift, under the pursuit condition. The proportions of the reported motion direction between the two viewing conditions did not coincide when they were plotted as a function of either the retinal displacement or of the objective displacement; however, they did coincide when plotted as a function of a mixture of the two. The result from Experiment 2 showed that the perceived jump size of the apparent motion was also dependent on both retinal and objective displacements. Our findings suggest that the detection of the apparent motion during smooth pursuit considers the retinal proximity and also the objective proximity. This mechanism may assist with the selection of a motion path that is more likely to occur in the real world and, therefore, be useful for ensuring perceptual stability during smooth pursuit.

Apparent motion is perceived motion that is produced by observing a display in which an element briefly appears at one location and then reappears at a different location. The spatiotemporal distance between elements is an essential factor for perceiving apparent motion and is known as the proximity. It has widely been believed that the computation for proximity is based on retinal displacement (e.g., Ullman, 1979). However, self-motion, such as eye movements, adds a motion vector to the retinal displacement.

Smooth pursuit eye movement allows to track moving objects of interest. Smooth pursuit can keep a tracked object in the fovea, but it adds a motion vector in the opposite direction of the pursuit to the retinal displacement. Usually, such additional motion vectors, introduced by pursuit, go mostly unnoticed. Extraretinal signals related to pursuit are taken into account to recover objective “world” motion during pursuit, as well as to transform retinal image motion into both head-centric and world-centric reference frames (e.g., Freeman, 2001; Freeman et al., 2009; Souman et al., 2006; Sperry, 1950; von Holst & Mittelstaedt, 1950; Wurtz, 2008). The most common explanation for this compensation is that the visual system integrates two velocities, one from visual inputs on the retina and the other from extraretinal information, such as an efference copy of oculomotor commands, at a higher level of motion processing (for review, see Furman & Gur, 2012; Spering & Montagnini, 2011; Wurtz, 2008). As a result of velocity integration, the perceptual velocity of the added motion vector is partially canceled out. Furthermore, when compared with the same stimuli during fixation, there is a reduction in both the contrast sensitivity for the pursuit-introduced retinal slip and the perceptual saliency of concomitant motion streaks, during smooth pursuit (Bedell & Lott, 1996; Schütz et al., 2007). These studies indicate that perception related to the pursuit-introduced motion vector is suppressed or eliminated.

In contrast, a recent study suggested that, in some cases, the pursuit-introduced motion vector may be positively enhanced (Terao et al., 2015). Specifically, motion perception along the direction opposite to that of eye movement was enhanced when a retinally rendered counterphase grating was presented during smooth pursuit (see also Terao et al., 2010 for a related motion-color effect). The aforementioned grating comprised two drifting components, one drifting along the opposite direction of the smooth pursuit and the other along the same direction as the smooth pursuit. This counterphase grating is almost always perceived to be moving opposite to the smooth pursuit, although the component gratings drift symmetrically on the retina. Terao et al. (2015) attributed this effect to the motion-signal enhancement along the antipursuit direction. Indeed, the motion bias due to the pursuit could be eliminated by reducing the contrast of the antipursuit direction relative to the pursuit direction, though this is not the sole explanation for this phenomenon. For example, the spatial coordinates on which the visual system detects motion may change from retinal to nonretinal coordinates (e.g., objective coordinates).

In this study, we replaced the continuous sinusoidal counterphase grating used by Terao et al. (2015) with the two-frame apparent motion of a square-wave grating. Even with no spatial shift on the screen between the two flashed gratings, pursuit at an appropriate speed and direction can introduce a shift so that the two gratings appear 180 degrees out of phase on the retina (Figure 1). Our preliminary observation with this stimulus showed that the direction opposite to the smooth-pursuit direction was always seen. This observation disagrees with the prediction by retinal proximity (Ullman, 1979), as retinal displacements are the same in both directions. However, it agrees with Terao et al. (2015), except now the motion is apparent motion. To explain the observed phenomenon, we can suggest two possible effects of smooth pursuit on the process of apparent motion. One is that smooth pursuit modulates the strengths of motion signals, enhancing the direction opposite to that of eye movement. The other, which can only be tested with apparent motion, is that the proximity that determines the strength of motion correspondence is computed based not only on the retinal proximity but also on the objective “world” proximity. These two possibilities can be dissociated by investigating the perceived direction of two-frame square-wave gratings at various phase shift angles.

**Figure 1.**
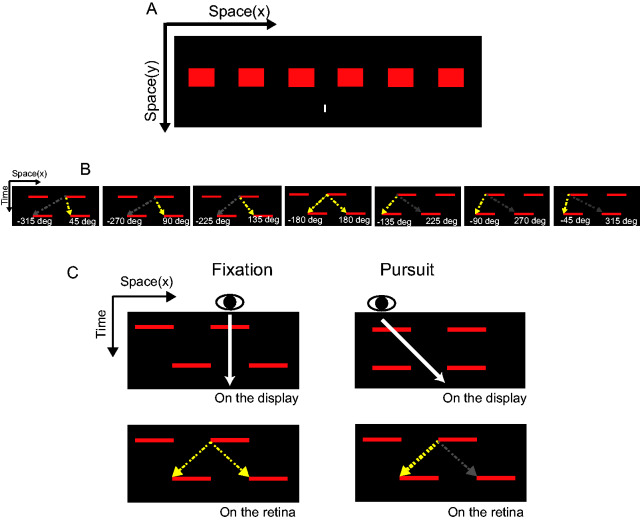
Experimental Setup. (A) The spatial configuration of the display comprises a square-wave grating and a white marker for pursuit or fixation. (B) Space–time plots of the apparent motion stimuli where the square-wave grating is flashed twice with a variety of phase shifts. The positive or negative values in each panel indicate the shift angle of the second grating in the rightward or leftward direction, respectively. In our experiment, we produced these seven phase shifts on the retina, but the patterns were the same on the objective “display” coordinate under the fixation condition. (C) The relationship between objective (top) and retinal (bottom) displacements for the fixation (left) and the pursuit (right) conditions. The white arrows indicate the eye position during fixation and the eye trajectory during rightward pursuit. During fixation, a phase shift of 180 degrees on the display corresponds to a phase shift of 180 degrees on the retina, and the perceived motion is ambiguous. During the pursuit to the right, the phase shift is 0 degree on the display but 180 degrees on the retina, and the dominant percept is a leftward movement direction.

In our experiments, we flashed a square-wave grating, comprising a horizontal array of bars, twice with a brief interval on a dark background (see Figure 1A). We varied the spatial phase of the square-wave grating, which could be described either with retinal coordinates or for the display with the objective coordinates. In either case, the spatial phase is defined as the shift angle of the second square wave from the first one, with the rightward direction being positive. According to the proximity principle of motion correspondence (Ullman, 1979), rightward motion is predominant when the retinal phase shift is smaller than 180 degrees, while leftward motion is predominant when the retinal phase shift is larger than 180 degrees. The frequencies of leftward and rightward motions are balanced when the phase angle is 180 degrees. Figure 1B depicts the seven different phase shifts on the retinal coordinates used in our experiment. We compared the perceived direction for the same retinal phase shift conditions between fixation and pursuit viewing conditions. Note that the objective phase shift angles were matched with the retinal ones under the fixation condition but were 180 degrees out of phase under the pursuit condition (see Figure 1C).

Figure 2 depicts how the two hypothetical effects of pursuit on apparent motion will affect the probability of the perceived motion direction, as a function of the retinal displacement. The black lines in Figure 2A and B indicate the predicted direction of perceived motion during fixation. According to the proximity principle of motion correspondence (Ullman, 1979), we expected that the probability of perceiving leftward motion during fixation would change in a sinusoidal fashion as a function of the retinal displacement, with a negative peak at 90 degrees and a positive peak at 270 degrees.

**Figure 2.**
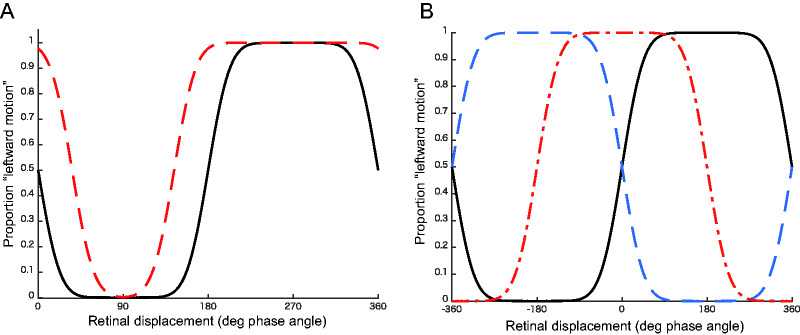
Changes in Perceived Direction Predicted by Two Possible Effects of Pursuit on Apparent Motion Direction. Pursuit direction is rightward. (A) The predicted enhancement of the motion signal along the direction opposite to the smooth-pursuit direction. The black line indicates the predicted probability for judging the motion as leftward during fixation as a function of the retinal displacement. The red line indicates the smooth pursuit condition. (B) The predicted situation in which the detection of the apparent motion during smooth pursuit is affected by objective displacement. The black line is the same as in (A). The red line indicates the predicted shift when the perceived motion direction is entirely determined by the objective displacement. The blue line indicates the predicted shift when the motion direction is determined by both retinal and objective displacements, with a 1:1 weighting ratio.

In Figure 2A, the red dashed line shows the prediction from the first hypothesis in which smooth pursuit enhances motion signals in the direction opposite to that of eye movement. In this case, the probability of perceiving leftward motion will remain dependent on the retinal phase shift but generally increase at any phase angle (vertical upward shift), except near the floor (0%) or ceiling (100%).

Figure 2B shows the prediction from the second hypothesis in which objective proximity affects the direction of apparent motion. In our experimental setup, the retinal displacement during pursuit was 180 degrees out of phase of the objective displacement (see Figure 1C). Thus, if the objective displacement determines the perceived direction of apparent motion entirely, the psychometric function, as a function of the “retinal” displacement, will horizontally shift 180 degrees (blue line). If the perceived direction of apparent motion is partially affected by the objective displacement, the psychometric function will shift horizontally in the opposite direction of smooth pursuit. The amount of shift depends on the degree of influence of the objective displacement. The red line in Figure 2B depicts the prediction of the case where the ratio of retinal versus objective influence is 1:1.

We tested these predictions in the following experiments.

## Experiment 1

### Methods

#### Observers

The observers in this study included two of the authors, M. T. and S. N., and three volunteers who were unaware of the aim of the experiments. All observers had normal or corrected-to-normal vision. They provided written informed consent before the experiments were conducted. The experiments and the consent form were approved by the NTT Communication Science Laboratories Research Ethics Committee, on the basis of the principles mentioned in the Declaration of Helsinki.

#### Apparatus

Visual stimuli were displayed on a cathode ray tube (CRT) monitor (GDM-F520, Sony, Inc., Tokyo, Japan) at a refresh rate of 120 Hz. The monitor was connected to a visual stimulus generator (VSG2/5, Cambridge Research Systems, Rochester, UK) installed in a workstation (Dell Precision 350, Dell, Inc., Round Rock, TX, USA). The monitor was calibrated using a colorimeter (Color Cal, Cambridge Research Systems) to linearize the gamma relationship. The spatial resolution of the monitor was 800 × 600 pixels, with each pixel subtending 1.5 minutes at a viewing distance of 113 cm. The observer sat with their head fixed on a chin rest and viewed the display binocularly. The room had no illumination, except for the stimulus presented on the CRT monitor. A keyboard was placed in front of the observer to record their responses. The movements of the dominant eye were monitored at 500 Hz using a video-based eye tracker (EyeLink II, SR Research, Ltd., Ottawa, ON, Canada). Analog output data obtained from the eye-tracker system were recorded on a disk for offline analysis, at a sampling rate of 1 kHz, using a data acquisition system (NR-2000; Keyence, Osaka-city, Osaka, Japan). To synchronize the timing of the stimulus presentation and eye data precisely, we chose a higher resampling rate than the original sampling rate of the eye tracker. In the offline analysis, the resampled eye position time series was low-pass filtered using a Butterworth filter with a cutoff frequency of 30 Hz.

#### Stimulus

The stimulus was a square-wave grating comprising a horizontal array of bars (see Figure 1A). Each array subtended 1.0 degree in height, 20 degrees in width, and 18 cd/m^2^. The background was a dark field. The height of each array, measured at the midline, was 1.5 degrees above the trajectory of the white marker. Notably, each bar subtended 1.52 deg in width and 1.0 degree in height.

#### Procedure

We tested three conditions: rightward pursuit condition, leftward pursuit condition, and fixation condition. Under the fixation condition, after the observer pressed a start key, the bar arrays were flashed twice on a dark background with an interval of 125 ms, while the observer fixated on the central stationary white marker. Under the pursuit condition, a key-press by the observer initiated the horizontal movement of the white point at a constant speed of 12.17 deg/s. The white marker moved a distance of 26.6 degrees either from the left end to the right end, or from the right end to the left end of the CRT screen. The observers were asked to pursue the white point as precisely as possible. When the marker approached the horizontal center of the screen, the square-wave grating was flashed twice with an interval of 125 ms when the marker reached the screen center. The procedure used for the leftward pursuit condition was similar to that used for the rightward pursuit condition, with the exception that all the stimuli were mirror reversed.

We manipulated the interframe retinal displacement by varying the spatial phase of the second square wave from the first square wave on the display. The spatial phase was defined as the shift angle of the second square wave in the rightward direction. Therefore, a small (large) phase angle resulted in a small (large) displacement in the rightward direction and a large (small) displacement in the leftward direction. Under the fixation condition, the following seven phase angles were presented: 45, 90, 135, 180, 225, 270, and 315 degrees. Under the pursuit condition, the display was 180 degrees out of phase with respect to the displacement between the two flashed gratings during the fixation condition. This manipulation was used to cancel out the 180 degree phase shift introduced by smooth pursuit (see Figure 1C). Consequently, the retinal displacement during the smooth pursuit condition was identical to that during the fixation condition, given perfect tracking.

All three conditions were tested in separate blocks that were presented in random order. The blocks were separated by an interblock rest interval of at least 5 minutes. The interframe displacement was randomly changed within each block. The task of the observer was to judge the motion direction of the grating (leftward or rightward). Note that the observers were not instructed regarding specific coordinates such as retinal or objective coordinates.

#### Eye-Movement Analysis

The eye-movement system was calibrated at the beginning of each block. The eye position data for each trial were stored on a disk for offline analysis. Eye velocity was obtained by the digital differentiation of the eye position with respect to time. To minimize the difference in the retinal image among the three stimulus conditions, we excluded trials in which the eye position shift between the two flashes exceeded the intended shift of 1.52 ± 0.13 deg. In a preliminary experiment, we examined how much any deviations from the expected pursuit would affect the performance and found that the effect of ±0.13 deg deviations was negligibly small.

The trials were also excluded if they contained rapid eye movements that exceeded 30 deg/s, which could indicate catch-up saccades. We collected data until at least 12 valid trials (typically 24) were obtained within the aforementioned eye-velocity criteria for each stimulus condition. The median eye velocity of the trials used for analysis, along with the quartile deviation, was 12.47 ± 0.648 deg/s.

### Results and Discussion

**Figure 3.**
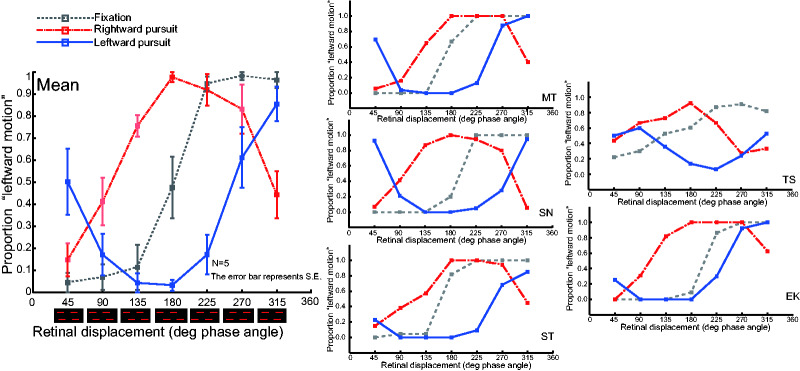
Results of Experiment 1. The perceived direction of motion is plotted as a function of the retinal displacement (quantified by calculating the degree of phase angle). The data were obtained for rightward smooth pursuit (red dash line), leftward smooth pursuit (blue line), and fixation (gray dots line). Individual panels correspond to mean data and each of the five observers.

Figure 3 depicts the mean and individual results in separate panels. The perceived motion direction under all conditions was plotted as a function of retinal displacement. Note that the retinal displacement of the fixation condition is in-phase with the objective displacement and that the retinal displacement of the pursuit conditions is 180 degrees out of phase with the objective displacement. The probabilities of the perceived motion direction for the fixation condition (gray dashed line in Figure 3) followed the standard proximity principle, as predicted in Figure 2 (gray dashed line). When the phase angle was 180 degrees, the perceived direction was ambiguous. The rightward motion predominated when the phase angle was smaller than 180 degrees, and the leftward motion predominated when the phase angle was larger than 180 degrees.

A systematic difference to the aforementioned trend emerged for the pursuit conditions. Here, the perceived direction was opposite to the pursuit direction when the phase angle was 180 degrees (blue and red lines in Figure 3). This result is consistent with previous research (Terao et al., 2015) as well as our preliminary observations. The probabilities of the perceived motion direction at other phase angles were different than the prediction from motion enhancement in the direction opposite to that of eye movement (Figure 2A). Instead, they are consistent with the hypothesis that objective proximity affects the direction of apparent motion (Figure 2B). In the mean data, the probability of seeing rightward motion increased at 225, 270, and 315 phase degrees in the rightward pursuit condition and at 45, 90, and 135 phase degrees in the leftward pursuit condition. The probability of seeing leftward motion increased at 45, 90, 135, and 180 phase degrees in the rightward pursuit and at 180, 225, 270, and 135 phase degrees in the leftward pursuit condition. In addition, the motion direction judged to be ambiguous was not at 180 degrees, but at ∼110 degrees and ∼290 degrees for the rightward pursuit condition. The effects of pursuit can be characterized by a horizontal shift of the psychometric function.

If the perceived motion direction was determined entirely by the objective proximity, the psychometric function plotted as a function of the retinal displacement would shift 180 phase degrees toward the direction opposite of eye movement. However, such a large shift was not observed. Our results seem to be consistent to the prediction that the perceived direction of apparent motion is partially affected by the objective proximity, as depicted by the red line in right panel of Figure 2B. Although the actual data (Figure 3) may look different from the sine-wave-based prediction, this is mainly because we did not collect any data at 0 and 360 degrees. The partial shift in the direction opposite to that of eye movement suggests that the direction of the apparent motion during smooth pursuit depends on both retinal and objective proximities.

## Experiment 2

The first experiment suggests that the visual system’s computation of motion correspondence during pursuit is based on the displacement of elements on a mixture of retinal and objective coordinates. To provide supportive evidence for this tentative conclusion, we additionally examined the perceived displacement, or jump size, in Experiment 2. If pursuit modulates the spatial coordinates, the apparent jump size may also change. Under fixation, where only retinal proximity matters, the physical jump size is largest at a 180 degrees phase shift, and accordingly, the perceptual jump size is expected to be largest at this phase shift. This is where the perceived motion direction is most ambiguous. If pursuit shifts the peak phase of the maximum motion ambiguity by changing the spatial coordinates, it may also shift the peak phase of the maximum perceived jump size in a similar way.

### Methods

The methods were identical to those in Experiment 1, except for the following. At the beginning of the experiment, the observers saw two reference stimuli while fixating: One was maximum displacement (180 degrees), and the other was minimum displacement (45 degrees or 315 degrees). The observers were instructed to assign 3 to the maximum displacement and 1 to the minimum displacement. In each experimental trial, participants were asked to rate the perceived jump size using 1, 2, or 3, in addition to judging the motion direction by pressing the corresponding key. Three observers participated in Experiment 2. They also participated in Experiment 1. One of them (S. T.) was naive to the aim of the experiment.

### Results and Discussion

**Figure 4.**
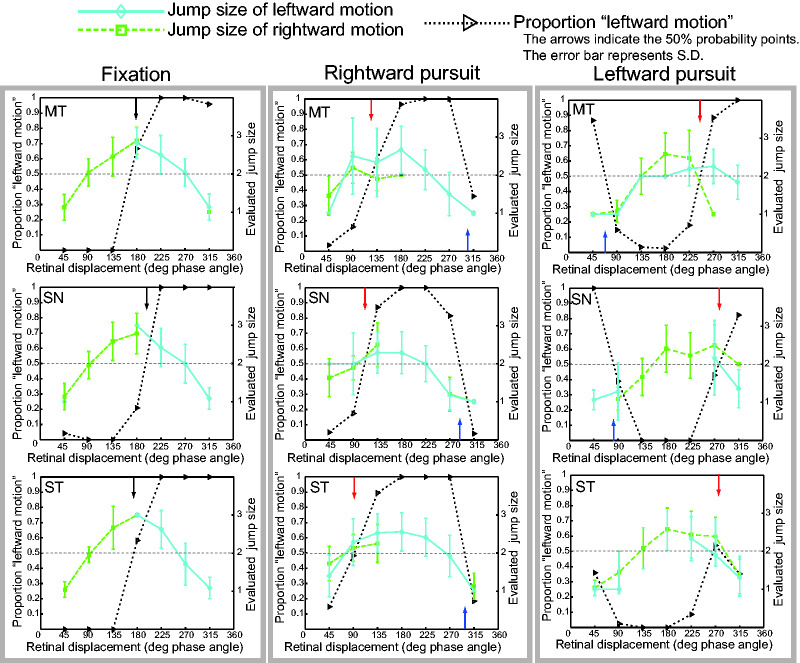
Results of Experiment 2. The proportion of seeing leftward motion direction and the reported perceived jump size are plotted as a function of the retinal displacement. The data were obtained for fixation (left column), rightward smooth pursuit (center column), and leftward smooth pursuit (right column). The gray short-dash line indicates the proportion of the leftward motion. The green long-dash line indicates the perceived jump size when the reported motion was leftward. The cyan line indicates the perceived jump size when the reported motion was rightward. The vertical axis on the left indicates the proportion of the leftward motion seen. The vertical axis on the right indicates the rating of the perceived jump size. The results of three observers for three viewing conditions are separately shown. The arrows indicate the phase angles of the maximal directional ambiguity (50% point) for fixation condition (black: positive slope) and for pursuit condition (red: positive slope, blue: negative slope).

Figure 4 depicts the proportion of the perceived motion direction and the evaluated jump size of the motion for each observer. The effects of pursuit on direction judgments show a similar pattern to Experiment 1. For the fixation condition, the curve of the perceived jump size shows a symmetric convex-upward function peaking at 180 degrees. The observers evaluated the apparent jump size to be maximum around the point of perceptual directional ambiguity (see black arrow in Figure 4). As the phase angle decreased or increased from 180 degrees, the estimated jump size gradually decreased. These results indicate that the observers were able to correctly report the jump size magnitude consistent with the retinal displacement under the fixation condition.

For the pursuit conditions, the curves of the perceived jump size appeared to shift leftward for rightward pursuit or rightward for leftward pursuit, away from the curve of the fixation condition. If pursuit changes the spatial coordinates for both motion direction computation and jump size estimation in a similar way, the phase angle of the maximum perceived jump size is expected to coincide with the phase angle of the maximum directional ambiguity on the positive slope (red arrow). The observed curve shifts were consistent with this prediction with respect to the shift direction, but the magnitude of the curve shift was not as large as predicted. This suggests that during smooth pursuit, the perceived jump size is affected not only by the retinal displacements but also by physical displacements, although the contribution of physical displacements to perceived jump size is not as large as it is to direction judgments.

## General Discussion

In this study, we considered the following two hypotheses regarding the determinant of the apparent motion direction during smooth pursuit. One is that the motion signals in the direction opposite to that of the smooth pursuit are enhanced. The other is that objective proximity affects the detection of apparent motion. To test which of these hypotheses is valid, we investigated the perceived direction of two-frame square-wave gratings with various spatial phases during smooth pursuit. In the case of the former hypothesis, the form of the psychometric function of smooth pursuit plotted against retinotopic displacement will be different from that of fixation. In the case of the latter hypothesis, the psychometric function of smooth pursuit will shift horizontally from the psychometric function of fixation. In Experiment 1, the behavior of the psychometric function of smooth pursuit was consistent with the prediction from the latter hypothesis. Specifically, the psychometric function of smooth pursuit appeared to shift from the psychometric function of fixation in the direction opposite to smooth pursuit. In Experiment 2, pursuit also produced horizontal shifts of the point of maximum perceived jump size, though the shift magnitudes were smaller than observed with the point of maximum directional ambiguity. The results from both experiments indicate that objective proximity affects the detection of apparent motion.

We showed that the psychometric function for motion direction shifted approximately 70 degrees toward the direction opposite to that of eye movement. This suggests that the direction of apparent motion during smooth pursuit was determined by 40% objective proximity and 60% retinal proximity, under the current experimental configuration (while the contribution ratio of the physical proximity may be smaller for jump size perception). This mixture ratio indicates that the visual system partially recovered the objective displacement to detect apparent motion. To recover the objective displacement, the visual system requires information regarding the motion vector that was added to the retinal displacement by smooth pursuit. Generally, extraretinal signals related to pursuit are considered to recover the objective world state during pursuit (e.g., Freeman, 2001; Freeman et al., 2009; Souman et al., 2006; Sperry, 1950; von Holst & Mittelstaedt, 1950; Wurtz, 2008). It is possible that the visual system takes extraretinal signals related to pursuit into account when estimating the additional motion vector by smooth pursuit. It is known that the additional motion vector by smooth pursuit is often underestimated in the case of the speed estimation process (see Freeman et al., 2010). Similarly, this underestimation might also occur in our phenomenon and might contribute to the partial recovery of objective displacement for the detection of apparent motion.

It is known that a mismatch between retinal and extraretinal signals leads to various perceptual phenomena. The Filehne illusion is a phenomenon in which an environmentally stationary object appears to move slightly in the opposite direction of pursuit (e.g., Filehne, 1922; Freeman & Banks, 1998; Mack & Herman, 1973; Wertheim, 1987). The Aubert–Fleischl phenomenon is a phenomenon in which a tracked object appears to move more slowly than when viewed during fixation (Aubert, 1886; Dichgans et al., 1975; Fleischl, 1882; Freeman & Banks, 1998; Wertheim & Van Gelder, 1990). These phenomena are considered products of imperfect velocity compensations caused by the integration of the underestimated pursuit velocity with the retinal velocity. Such errors in the velocity integration process may modify the apparent velocity of motion but will not change the pattern of motion correspondence. Thus, our effect and these classical phenomena presumably have different mechanisms, even though both phenomena might result from a mismatch between retinal and extraretinal signals.

The mismatch between retinal and extraretinal signals also causes localization errors (for review, see Schlag & Schlag-Rey, 2002). One might consider that our findings are related to the localization errors for shortly presented objects during smooth pursuit, given that proximity computation is based on the position information. However, this is likely because the mislocalization during pursuit has been ascribed to visual neural delays, which make visual information of an object combined with eye position information at different moments (e.g., Brenner et al., 2001). This mismatch presumably affects the two flashed gratings in our experiments in the same manner and thus produces no relative phase shift change between them.

Similarly, spatial and temporal properties of perception, memory, and representational momentum are suggested to contribute to localization errors during smooth pursuit (e.g., Brenner et al., 2001; Freyd & Finke, 1984; Kerzel, 2000, 2005; Rotman et al., 2004), but they are unlikely to be responsible for our effect because they will affect the two gratings used in our experiments in the same manner.

Although this study was motivated by the findings of Terao et al. (2015), it remains controversial whether the contribution of objective proximity can also explain the motion direction perceived for the counterphase grating during pursuit. In our preliminary observation, we did not find a similar slower (shorter-displacement) bias in the judgment of the continuous counterphase grating direction. In other words, the direction of the slower component was not always judged as the dominant direction when two continuous drifting gratings along mutually opposite directions moving at different speeds were superimposed. Instead, two opposing motions were seen simultaneously for most of the time. Further investigation is necessary to clarify this issue.

Motion detection considering objective proximity during smooth pursuit may serve a useful function to ensure perceptual stability. Proximity, which is the preference to choose a smaller displacement, is an essential cue for the motion-correspondence problem. The motion-correspondence problem is a problem in which the visual system must determine which elements originate from the motion of which object in the world when multiple elements are presented in an apparent motion display. The elements that are close to each other have a stronger affinity than those that are farther apart. It has been considered that proximity computation is based on retinotopic displacement (Ullman, 1979). However, the proximity in the retinotopic displacement is useless for precisely estimating the object motion during eye movements, as the proximity in retinal coordinates does not reflect a physical one because of eye movements. If the proximity computation depends entirely on the proximity of the retinal image during eye movements, false matching might occur. Upon determining the matching of the motion path, it is almost impossible to rematch the motion path at a subsequent processing stage. Accordingly, the retinal proximity should be compensated at an early motion-processing stage as much as possible to achieve precise object-motion estimation. Our results indicate that the matching of motion path during smooth pursuits is determined not only by the retinal proximity but also by the objective proximity. Here, the objective proximity presumably helps reduce the false matching of motion paths. In addition, it may serve as a useful function for reducing image degradation due to motion. The neural integration of visual signals along the trajectory of moving objects can reduce motion-blur perception (Burr, 1981; Burr & Ross, 1986; Nishida, 2004; Nishida et al., 2007). This is because neural integration prevents signal mixtures among spatially adjacent inputs that induce image degradation (Terao et al., 2010; Watanabe & Nishida, 2007). Given that considering the objective proximity helps to obtain motion that might occur in the environment, the visual signals along the motion trajectory would be effectively integrated, and accordingly, we may obtain an impression of clear vision despite eye movements.

To summarize, we demonstrated that objective proximity affects the detection of apparent motion during smooth pursuit. Motion detection considering the objective displacement helps select a motion path that is more likely to occur in the real world and, therefore, may be useful for ensuring perceptual stability during smooth pursuit.
